# Complete Genome Sequence of the Broad-Host-Range *Paenibacillus larvae* Phage phiIBB_Pl23

**DOI:** 10.1128/genomeA.00438-13

**Published:** 2013-09-05

**Authors:** Ana Oliveira, Luís D. R. Melo, Andrew M. Kropinski, Joana Azeredo

**Affiliations:** Institute for Biotechnology and Bioengineering, Centre of Biological Engineering, Universidade do Minho, Campus de Gualtar, Braga, Portugala; Public Health Agency of Canada, Laboratory for Foodborne Zoonoses, Guelph, ON, Canadab; Department of Molecular and Cellular Biology, University of Guelph, Guelph, ON, Canadac

## Abstract

*Paenibacillus larvae* is a Gram-positive bacterium that causes American foulbrood, an important disease in apiculture. We report the first complete genome sequence of a *P. larvae* phage, phiIBB_Pl23, isolated from a hive in northern Portugal. This phage belongs to the family *Siphoviridae*.

## GENOME ANNOUNCEMENT

American foulbrood is a bacterial disease caused by *Paenibacillus larvae*, a Gram-positive bacterium wherein the spore is the infectious form. *P. larvae* causes hive destruction and consequently important economic losses in apiculture worldwide (1). Moreover, a European Community regulation (no. 2377/90) limits the presence of antibiotics in honey, excluding its use for therapy. Therefore, the development of alternative antimicrobial methods is of utmost importance. Bacteriophages, viruses that infect and lyse bacteria, have shown great efficacy in controlling bacterial diseases in animal production (2–5).

The phage phiIBB_Pl23 was isolated after culturing of strain H23 (host) found in honeybee larvae from a Portuguese hive. This phage formed plaques on most of the *P. larvae* strains tested. Morphologically, it is from the *Siphoviridae* family.

PhiIBB_Pl23 was propagated in H23 under incubation at 37°C with 5% CO_2_, and afterward the phage DNA was extracted. The genome was sequenced using Roche/454-recommended procedures at the Plateforme d’analyses génomiques of the Institut de Biologie Intégrative et des Systèmes (Laval University, Québec, Canada). Shotgun reads were assembled using the gsAssembler module of Newbler v 2.5.3. Potential open reading frames (ORFs) were annotated using myRAST (6). The presence of Shine-Dalgarno sequences upstream of each ORF and the search for additional ORFs were checked manually in Kodon (Applied Maths, Austin, TX). Putative protein functions were assigned using BLASTP (7) and Pfam (8) with databases available on April 2013. Transmembrane domains were predicted using Phobius (9) and TMHMM (10). Putative host-dependent (SigA) promoters were discovered with their consensus sequence TTGACA-N14-tgnTATAAT (11). The Rho-independent terminators and calculations of the free energy of their secondary structures were predicted using ARNold (12) and Mfold (13), respectively. tRNAscan-SE (14) and ARAGORN (15) were used for tRNA detection.

The genomic double-stranded DNA of phiIBB_Pl23 consists of 41,294 bp, with a GC content of 40.9%. The latter value is less than that for the host at 44 GC mol%. The genome was scanned for coding DNA sequences (CDS) resulting in 68 CDS, ranging from 117 bp (39 codons) to 2,928 bp (976 codons). The initiation codon of 81% of the predicted CDS is ATG, while 10% of the CDS start with GTG and 9% with TTG. Based on BLAST and Pfam analysis, 51% of the proteins have been assigned functions; 18% were defined as conserved hypothetical proteins and 31% are unique. The presence of a serine recombinase/resolvase (gp30), repressor (gp31), and antirepressor (gp39) indicates that this is a temperate phage (16). The endolysin of this phage is an *N*-acetylmuramoyl-l-alanine amidase, identified as an amidase_2 domain, common to other *Bacillus* spp. (17). It also encodes a 975-amino-acid protein (gp26) possessing several ricin-type beta-trefoil lectin domain-like domains (pfam14200) identical to *Paenibacillus larvae* toxin 1 (AGJ74029), suggesting that like many temperate phages, phiIBB_Pl23 is capable of lysogenic conversion (18, 19).

Two putative promoters were identified as having homology with SigA *Bacillus* promoters and three rho-independent terminators but no tRNAs.

### Nucleotide sequence accession number.

This whole genome shotgun project has been deposited at GenBank under the accession no. KF010834. The version described in this paper is the first version.
